# *R-spondin1 *and *FOXL2 *act into two distinct cellular types during goat ovarian differentiation

**DOI:** 10.1186/1471-213X-8-36

**Published:** 2008-04-02

**Authors:** Ayhan Kocer, Iris Pinheiro, Maëlle Pannetier, Lauriane Renault, Pietro Parma, Orietta Radi, Kyung-Ah Kim, Giovanna Camerino, Eric Pailhoux

**Affiliations:** 1 INRA, UMR 1198, ENVA, CNRS, FRE 2857, Biologie du Développement et de la Reproduction, Jouy-en-Josas, F-78350, France; 2 Dipartimento di Patologica Umana ed Ereditaria, Università di Pavia, Via Forlanini 14, 27100 Pavia, Italy; 3 Nuvelo, Inc., 675 Almanor Avenue, Sunnyvale, CA 94085, USA

## Abstract

**Background:**

Up to now, two loci have been involved in XX sex-reversal in mammals following loss-of-function mutations, PIS (Polled Intersex Syndrome) in goats and *R-spondin1 *(*RSPO1*) in humans. Here, we analyze the possible interaction between these two factors during goat gonad development. Furthermore, since functional redundancy between different *R-spondins *may influence gonad development, we also studied the expression patterns of *RSPO2, 3 *and *4*.

**Results:**

Similarly to the mouse, *RSPO1 *shows a sex-dimorphic expression pattern during goat gonad development with higher levels in the ovaries. Interestingly, the PIS mutation does not seem to influence its level of expression. Moreover, using an RSPO1 specific antibody, the RSPO1 protein was localized in the cortical area of early differentiating ovaries (36 and 40 d*pc*). This cortical area contains the majority of germ cell that are surrounded by FOXL2 negative somatic cells. At latter stages (50 and 60 d*pc*) RSPO1 protein remains specifically localized on the germ cell membranes. Interestingly, a time-specific relocation of RSPO1 on the germ cell membrane was noticed, moving from a uniform distribution at 40 d*pc *to a punctuated staining before and during meiosis (50 and 60 d*pc *respectively). Interestingly, also *RSPO2 *and *RSPO4 *show a sex-dimorphic expression pattern with higher levels in the ovaries. Although *RSPO4 *was found to be faintly and belatedly expressed, the expression of *RSPO2 *increases at the crucial 36 d*pc *stage, as does that of *FOXL2*. Importantly, *RSPO2 *expression appears dramatically decreased in XX PIS^-/- ^gonads at all three tested stages (36, 40 and 50 d*pc*).

**Conclusion:**

During goat ovarian development, the pattern of expression of *RSPO1 *is in agreement with its possible anti-testis function but is not influenced by the PIS mutation. Moreover, our data suggest that RSPO1 may be associated with germ cell development and meiosis. Interestingly, another RSPO gene, RSPO2 shows a sex-dimorphic pattern of expression that is dramatically influenced by the PIS mutation.

## Background

In most mammals, gonad differentiation depends on the presence or absence of the *SRY *gene, the sex-determining region of Y chromosome [1]. *SRY *encodes an HMG domain protein that is sufficient to switch on the testis-differentiating pathway [2-4]. Following *SRY *expression, specifically in the future supporting cells of the XY bipotential gonad, *SOX9 *remains up-regulated and Sertoli cell differentiation occurs [5]. The crucial role of *SOX9 *in testis differentiation has been demonstrated by gain and loss of function mutations in mice [6-9]. Acting with the transcription factor *SOX9 *is the diffusible growth factor *FGF9 *shown to be involved in *SOX9 *expressional maintenance, XY-specific cell migration from the mesonephros into the gonad and germ cell survival in the fetal testis [10-12].

In the XX female counterpart, the repression of the testis-differentiating pathway seems to be part of the molecular events leading to ovarian differentiation. Indeed, in human and domestic animals, XX male phenotypes can result from homozygous mutations in anti-testis genes specifically expressed in ovaries [13]. In mouse, the secreted protein Wnt4 antagonizes the testis specific genes *Sox9/Fgf9*, behaving as an anti-testis molecule [10]. Although some male features appear in XX *Wnt4*^-/- ^mice, such as a testis-specific coelomic vessel and testosterone production, XX sex-reversal is not completely achieved [14,15]. Up to now, two loci have been proved to be associated with complete XX sex-reversal, PIS in goat [16] and more recently, *RSPO1 *in human [17].

In goat, the PIS (Polled Intersex Syndrome) mutation gives rise to an absence of horns in heterozygous and homozygous animals of both sexes (dominant trait) and to a female-to-male sex-reversal in XX homozygous animals (recessive trait) [18]. Testis differentiation in XX PIS^-/- ^animals occurs very precociously during fetal life (*SOX9 *and *AMH *up-regulation occur between 36 and 40 d*pc*), with a ~4 days delay compared to XY males and affects primarily the supporting cells of the gonads [19]. In normal XY gonads, *SOX9 *and *AMH *up-regulation occur between 34 and 36 d*pc*. During this period, *FOXL2 *and *CYP19 *transcriptions begin to be detectable in normal XX gonads [19]. From a genetic point of view, this goat syndrome is due to an autosomal deletion of 11.7 kb localized on goat chromosome 1 [16,20]. This deletion encompasses elements enhancing the ovarian transcription of at least three genes: *FOXL2 *encoding a transcription factor, *PFOXic *and *PISRT1*, two non-coding RNAs [21,22]. All these genes are expressed from the first stage of ovarian differentiation to adulthood in normal XX PIS^+/+ ^and PIS^+/- ^gonads. Their expression is lost in XX PIS^-/- ^sex-reversed gonads [16,22]. Interestingly, XX *Foxl2*^-/- ^mice show an early disruption of folliculogenesis that recapitulates a phenotype of Premature Ovarian Failure (POF) also encountered in XX BPES type I patients (Blepharophimosis Ptosis Epicanthus inversus Syndrome, MIM#110100) carrying a heterozygous mutation of the *FOXL2 *gene [23-26]. The phenotype discrepancy of the ovarian *FOXL2/Foxl2 *loss of function mutations between goat and mouse can be either due to the complexity of the goat PIS mutation that affects other genes than *FOXL2 *or to species-specific differences in the ovarian differentiating pathway.

More recently in human, homozygous mutations of the *R-spondin1 *gene (*RSPO1*) have been shown to be responsible for XX sex-reversal associated with skin defects, palmoplantar hyperkeratosis (PPK) and predisposition to squamous cell carcinoma (SCC) [17]. *R-spondin1 *is a member of a recently identified small gene family of secreted molecules potentially acting via the *FZD/LRP *receptors (Frizzled/LDL receptor-related protein); a pathway previously described for the WNT secreted factors [27,28]. Binding to these *FZD/LRP *receptors induces beta-catenin accumulation in the cytoplasm and eventually its nuclear translocation where it is engaged in gene activation through its association with T-cell factor (TCF) transcription factors [29].

In this study, the expression profiles of the four R-spondin members was determined during the development of goat gonads. Their expression was also assessed in XX sex-reversed PIS^-/- ^gonads at different developmental stages. Surprisingly, expression of *RSPO2 *seems more sensitive than that of *RSPO1 *to PIS sex-reversal. Moreover, using a RSPO1-specific antibody [30], we show that goat RSPO1 is localized in the cortical part of the developing ovary where most of the germ cells lies.

## Results

### Goat *RSPO1 *cDNA characterization and phylogenic conservation

**Figure 1:**
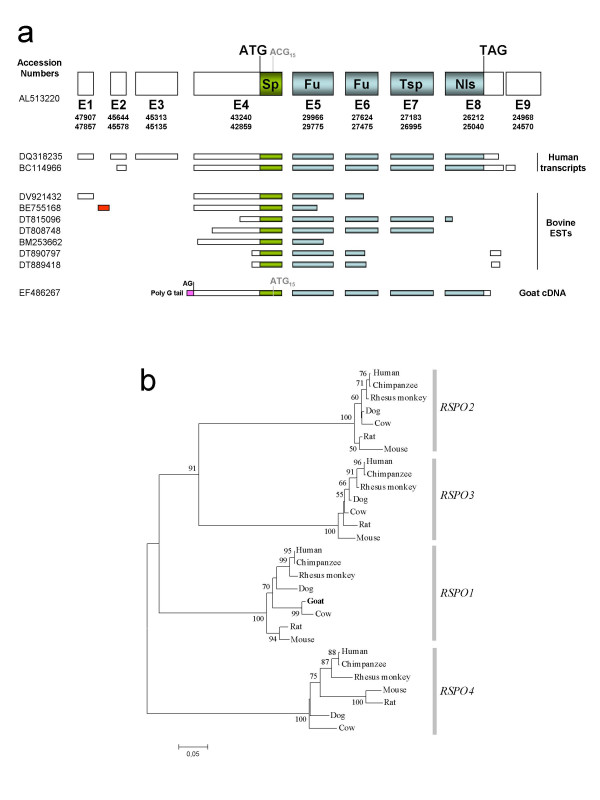
**Goat RSPO1 characterization and phylogeny**. a) Goat *RSPO1 *cDNA structure is compared with seven *RSPO1 *bovine Expressed Sequence Tag (EST), two human transcripts and the human gene. Genbank accession numbers of the different sequences are given on the left. A bovine-specific 5' non-coding exon 1 is depicted in red. The goat-specific part of exon 1 is depicted in pink. In goat, the second ATG (ATG_15_) is not conserved in human (ACG_15_). Sp = Signal peptide; Fu = Furin domain; Tsp = Thrombospondin domain; Nls = Nuclear localization signal; AG = conserved acceptor spicing site. b) A Neighbor-Joining tree was constructed with 28 DNA sequences from the four *RSPO *genes belonging to seven mammalian species, plus the putative goat *RSPO1 *ORF (the corresponding goat sequence is depicted in bold). Confidence values (higher than 50%) after bootstrap test are shown at each node. Genbank accession numbers are given in the methods section.

The entire open reading frame of goat *RSPO1 *cDNA [GenBank: EF486267] has been isolated by RT-PCR with primers (Spondin-ATG1bis and Spondin-TAG) derived from regions of the bovine sequence that are highly conserved in human. In order to determine the initiation start site and the promoter used in the female gonad, 5'-RACE experiments were carried out on 45 d*pc *goat ovaries mRNA. A unique initiation site has been found 349 bp upstream of the conserved ATG initiator codon (Fig. 1a). This initiation start site lies just downstream of a 10-bp polyG-tail that is present in goat genomic DNA [GenBank: EF486271] and no transcript 5' to this polyG-tail were detected despite several attempts. Thus, surprisingly, the goat ovarian transcript does not use the conserved AG acceptor splicing site, located 303 bp upstream of the ATG as observed in bovine (n = 2) and human (n = 2) (Fig. 1a). Consequently, in 45 d*pc *goat ovaries the first exon of the *RSPO1 *gene includes the ATG initiator codon, whereas in human and bovine, the exon encompassing the first codon can be exon2, 3 or 4, depending on the number of *RSPO1 *untranslated 5' exons (Fig. 1a). In conclusion, the precise location of the promoter of the *RSPO1 *gene appears to be variable due to the potential existence of different tissue-specific promoting regions [see Additional file 1].

In order to confirm that the goat *RSPO1 *cDNA sequence corresponds to the correct *R-spondin *gene, a phylogenetic tree was built with several sequences of the *R-spondin *family belonging to different mammalian species. The NJ tree built with the four *R-spondin *genes, belonging to seven mammalian species, is depicted in Fig. 1b. As expected, the goat *RSPO1 *sequence characterized in this work, belongs to the *RSPO1 *cluster, being more closely related with the bovine sequence.

The sequence of the PCR products obtained for the three other *R-spondin *genes have been determined and compared with the bovine and human genomes to ascertain their affiliation [GenBank: EF486268, EF486269, EF486270, for *RSPO2*, *3 *and *4*, respectively].

### *R-Spondin *gene expression in normal goat gonad development

**Figure 2:**
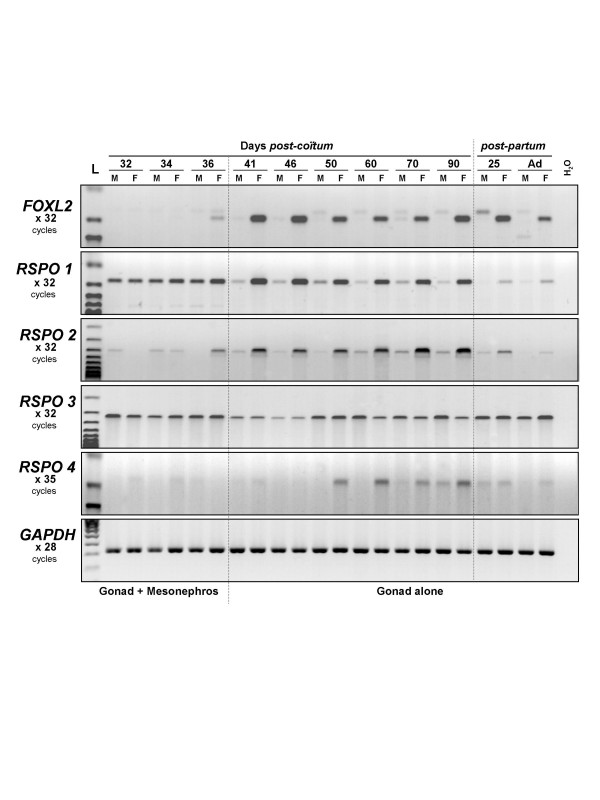
**RT-PCR expression analyses of *R-spondin *genes during normal goat gonadal development**. Expression profiles of *FOXL2 *and the four *R-spondin *genes were determined at 11 developmental stages (9 fetal stages and 2 stages after birth), in both sexes (M = male, F = female). *GAPDH *gene was used as control. The name of the amplified gene and the number of PCR cycles done is given on the left margin. L: 100 bp DNA ladder molecular weight marker (Bioline).

*R-spondin *gene expression profiles were determined during goat gonad development by RT-PCR with specific primers (Fig. 2 and Table 1). Interestingly, both *RSPO1 *and *RSPO2 *show a female-specific expressional profile from the crucial 36 d*pc *stage to adulthood, as observed for *FOXL2*. However, some slight differences exist between *RSPO1 *and *RSPO2 *gene regulation. Firstly, each gene reaches its higher level of expression at different developmental stages, before germ cell meiosis (55 dpc) for *RSPO1 *and at the time of follicle formation for *RSPO2 *(from 70 dpc until before birth). Secondly, in contrast to what observed in mice, our data suggest that *RSPO1 *is also expressed in both male and female mesonephroi [see Additional files 1 and 2].

**Table 1:** Primers and PCR conditions

**Experiment**	**Primer names**	**Primer sequences**	**Temp./MgCl2**
RT-PCR	Spondin-1	5'-CTGCGAGGCCTGCTTCAGCC-3'	58°C/1,5 mM
	Spondin-3	5'-GGAGCAAGGCCCCCACAGAG-3'	
	RSPO2-1F	5'-ATGGATTACAGCCASTGCCAAG-3'	58°C/2,5 mM
	RSPO2-2R	5'-TGCCGTGTTCTGGTTTCCAGAC-3'	
	RSPO3-1F	5'-AATACATYGGCAGCCAAAACGCC-3'	59°C/2,0 mM
	RSPO3-2R	5'-TGTCAAGGCACTTTCCAAGGTG-3'	
	RSPO4-3F	5'-TATCCGCCAGTACGGCAAGTG-3'	57°C/2,5 mM
	RSPO4-4R	5'-CCCTTGTACAGGTAAAACCGCC-3'	
	FOXL2-9	5'-GGCCCCCTGAGCCAGCGCCC-3'	58°C/1,5 mM
	FOXL2-10	5'-CCCCGACGCTGAGGTGCCCG-3'	
	GAPDHs	5'-AGGCCATCACCATCTTCCAG-3'	58°C/1,5 mM
	GAPDHas	5'-GGCGTTGGACAGTGGTCATAA-3'	
5'RACE	Spondin-2	5'-CCCTCCGGAAGCCACAGAGC-3'	58°C/1,5 mM
	Spondin-5	5'-GCCTTTGGCACAGGCCTGGC-3'	
	Spondin-6	5'-AGGATGGCAAGCAGACGCCC-3'	
pSG5 constructs	Spondin-ATG1bis	5'-CCGGCGAGTGACTATGCGGC-3'	60°C/1,5 mM
	Spondin-ATG2	5'-GTGTGGTGGCCTTGGTTCTG-3'	
	Spondin-TGA	5'-TCGCATGGACCGGGAGGCTG-3'	
*In situ *hybridization	Spondin-12	5'-CCATCTGGGAGAGGTAGAATC-3'	55°C/1,5mM
	Spondin-13	5'-CACCTCCTCTGAAAAACTTCCC-3'	
	HIS-RSPO1-1F	5'-GGCGCTCGAAGACGCAAGGG-3'	58°C/1,5 mM
	HIS-RSPO1-2R	5'-GGATAAAGTCACACAGCTGG-3'	

*RSPO3 *is similarly expressed in XX and XY gonads while *RSPO4 *was faintly expressed from 50 to 90 dpc, with a female-specific sex-dimorphic pattern.

### *R-Spondin *gene expression in XX sex-reversed goat gonads

In order to understand if the PIS mutation affects the pattern of expression of *RSPO1 *or of the other *R-spondin *genes during gonadal differentiation, the expression profiles of the four *R-spondin *genes were determined in sex-reversed versus normal gonads. We have previously shown that the crucial stages in the goat sex-reversal pathology are between 36 and 40 d*pc *[19]. Briefly, at 36 d*pc*, even if no sex-reversal defect can be noticed by histology on XX PIS^-/- ^gonads, a dramatic decrease of *CYP19 *gene expression is detectable. *CYP19 *has been identified as a direct ovarian-specific target of *FOXL2 *[31]. At 40 d*pc*, *SOX9 *and *AMH *expressions have increased and masculinization is clearly detectable by histology [19].

**Figure 3:**
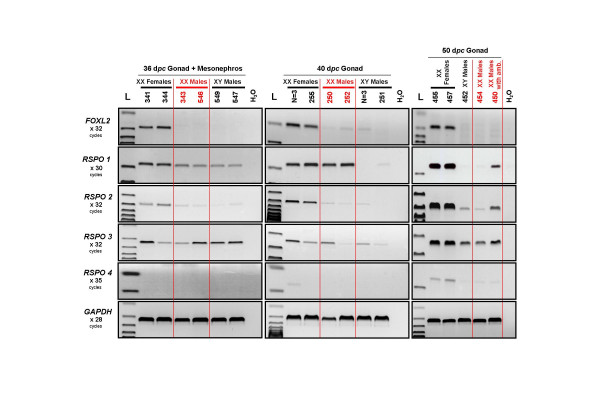
**RT-PCR expression analyses of *R-spondin *genes in XX PIS^-/- ^sex-reversed goat gonads**. Expression profiles of *FOXL2 *and the four *R-spondin *genes were determined at 3 developmental stages (36, 40 and 50 d*pc*) in XX sex-reversed gonads (XX Males) in comparison with normal males and females ones. GAPDH was used as control. The name of the amplified gene and the number of PCR cycles done is given on the left margin. Samples are from individual fetuses (identified by their number) or from a pool of 3 gonads (N = 3). L: 100 bp DNA ladder molecular weight marker (Bioline).

Interestingly, even if *RSPO1 *expression seems to be slightly decreased in XX PIS^-/- ^gonads at 36 d*pc*, its level of expression in XX sex-reversed gonads remains comparable to that seen in normal female at 40 d*pc *(Fig. 3). In contrast, *RSPO2 *expression appears to be significantly lower in XX sex-reversed gonads compared to XX normal ovaries at all three tested stages (36, 40 and 50 d*pc*). Indeed, *RSPO2 *mRNA levels are similar to those detected in XY control male testes (Fig. 3). Interestingly, at 50 d*pc*, a faint but higher mRNA levels of *RSPO1 *and *RSPO2 *can be detected in sex-reversed gonads from XX male with genital ambiguities (N°450) as compared to those of fully masculinized XX or XY males. Although 50% of XX PIS^-/- ^sex-reversed gonad develops as testes inducing complete masculinization of the external genitalia, the remaining 50% develops as ovo-testis associated with external genital ambiguities [19,32,33]. It is thus tempting to speculate that the remaining detectable *RSPO1 *and *RSPO2 *expression comes from the ovarian part of these hermaphrodite XX PIS^-/- ^gonads.

As described above, *RSPO3 *appears to be ubiquitously expressed and not affected by the PIS mutation. At 50 d*pc*, *RSPO4 *begins to be very faintly expressed specifically in normal ovaries, but not in XX sex-reversed or XY testes (Fig. 3).

### Immunodetection of RSPO1 protein in transfected COS7 cells

**Figure 4:**
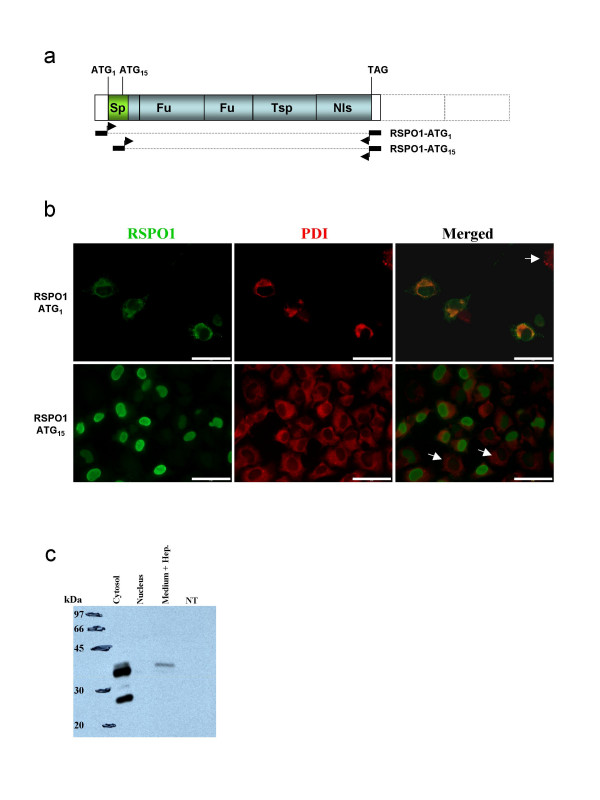
**Test of the *RSPO1 *antibody specificity by immuno-florescence studies and Western blotting**. a) Schematic representation of goat RSPO1 cDNAs used in transfection experiments in COS7 cells. The longest comprise the first initiator codon (ATG_1_). The smallest encodes a putative protein beginning at ATG_15_. The black rectangles with arrows depicted the location of the primers used in order to clone both cDNAs. b) RSPO1 and Protein Disulphide Isomerase (PDI) immuno-detection in COS7 cells transfected with RSPO1-ATG_1 _or RSPO1-ATG_15 _expression vectors. Scale bars = 5 μm. c) Western blot detection of RSPO1 on total proteins extracted from the cytosolic or nuclear compartments and from the heparin-supplemented culture medium of COS7 cells transfected with RSPO1-ATG_1 _compared with none transfected cells (NT).

*RSPO1 *transcript analysis revealed that goat mRNA contains two possible starting codons (ATG_1 _and ATG_15_) that may produce proteins with or without a signal peptide, respectively. In order to establish the stability and cellular sub-localization of the two putative proteins, we produced two constructs, one containing both potential initiation codons (ATG_1 _and ATG_15_), and the other retaining only the ATG_15 _(Fig. 4a). In order to evaluate the specificity of anti-hRSPO1 (Nu-206) antibody (provided by Dr K-A Kim) in goat, both immunohistochemistry and Western blot were performed on COS7 cells transfected (or not) with RSPO1 cDNA constructs (Fig. 4).

Nu-206 anti-hRSPO1 antibody appeared to be highly specific since no signal was detected in non-transfected cells (Fig. 4b, 4c). Double immunofluorescence studies were carried out for RSPO1 and PDI (Protein Disulphide Isomerase), an abundant soluble resident protein of the endoplasmic reticulum (ER) [34]. As expected, the full-length protein RSPO1-ATG_1 _was localized both in the ER with PDI and on the cell membrane (Fig. 4b). When the first ATG codon was missing (RSPO1-ATG_15_) the resulting protein was detected in the nucleus, highlighting the efficiency of the putative nuclear localization signal (Fig. 4b). By Western blot analyses, a 25 kDa protein was detected as expected on protein extracts of COS7 cell transfected with the full-lenght *RSPO1 *cDNA (Fig. 4c). Bands of higher molecular mass were also visible suggesting post-translational modifications of the protein. Moreover, the RSPO1-ATG_1 _protein was detected in the culture medium when heparin is added, and the secreted form corresponds to the most post-translationally modified one (highest molecular mass).

### Ovarian localization of RSPO1 at different developmental stages

**Figure 5:**
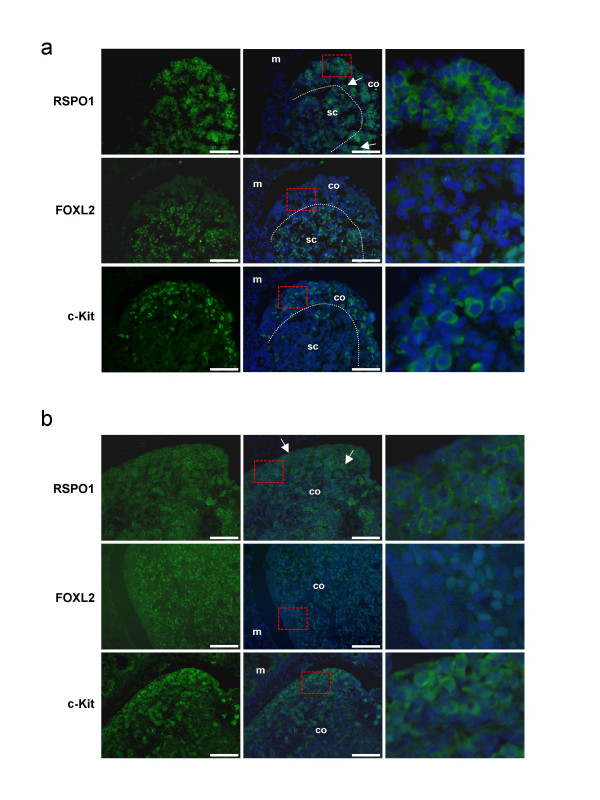
**RSPO1, FOXL2 and c-Kit immuno-detection on goat ovaries**. a) 40 d*pc *ovaries. b) 50 d*pc *ovaries. Scale bars: 100 μm. The fluorescent staining is presented alone (left column) or with a DAPI blue nuclear-specific counterstaining (medium and right columns). The right column corresponds to a 5.0 enlargement of the red rectangle depicted on the medium column. At 40 d*pc*, RSPO1 is detected in the cortical area (co) of the ovaries where most of c-Kit positive germ cells lies. At this stage both somatic and germ cells are stained (arrows show 2 germinal cells). By contrast, FOXL2 positive cells are in the sub-cortical area (sc) of these early developing ovaries. At 50 d*pc*, RSPO1 is detected mainly around the c-Kit positive germ cells easily recognizable by their large and round nuclei (arrows). At this stage, FOXL2 positive somatic cells are located in the two ovarian compartments, cortex and medulla. m = mesonephros; the dotted line delimits both areas (co and sc).

**Figure 6:**
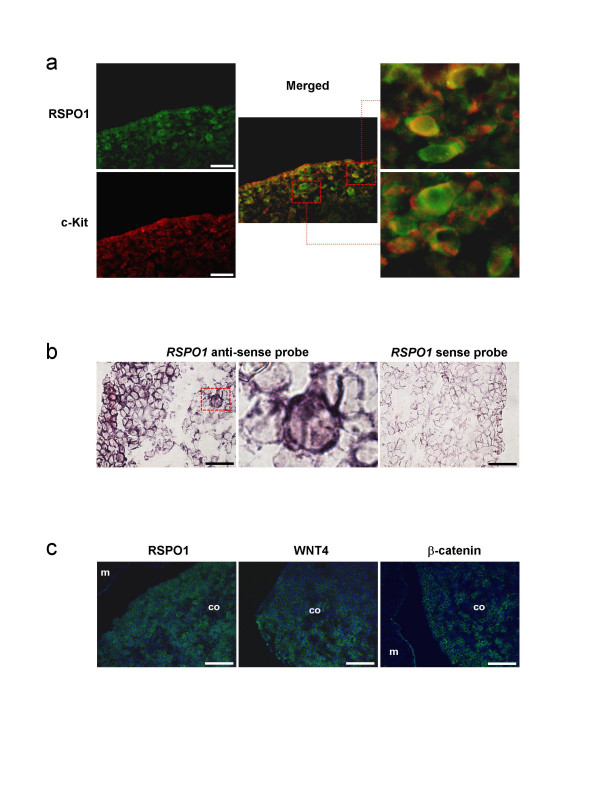
**RSPO1, FOXL2, c-Kit, WNT4, β-catenin immuno-detection and RSPO1 *in situ *hybridization on goat ovaries**. a) Double immuno-detection of RSPO1 (green) and c-Kit (red) on 40 d*pc *ovaries, showing a co-localization of both proteins on germ cell membranes. The right pictures correspond to a 5.0 enlargement of the red rectangles depicted on the medium picture. Scale bars: 50 μm. b) *In situ *hybridization of *RSPO1 *specific probes on 45 d*pc *ovaries. The medium picture correspond to a 5.0 enlargement of the red rectangles depicted on the left picture, showing a positive germ cell. Note the great majority of cortical cells (somatic + germinal) expressed RSPO1. Scale bars: 25 μm. c) Immuno-detection of RSPO1, WNT4 and b-catenin on 50 d*pc *ovaries. Note the similar localization of these 3 proteins, mainly on germ cells. m = mesonephros; co = cortex; Scale bars: 100 μm.

**Figure 7:**
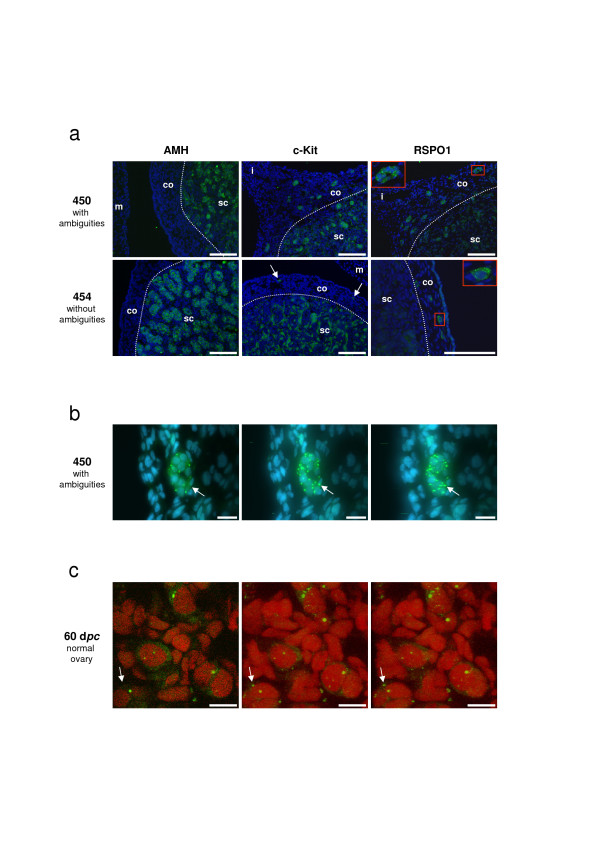
**RSPO1, AMH and c-Kit immuno-detection on goat sex-reversed gonads at 50 d*pc***. The fluorescent staining is presented with a DAPI blue (a, b) or a propidium iodide (c) nuclear-specific counterstaining. a) AMH (Sertoli-specific cell marker) and c-Kit (Leydig and germ cells marker) detections reveal the difference in testis development between XX male with ambiguities (N°450) and without ambiguity (N°454). In both cases, only the germ cells located in the cortical region (co) outside the seminiferous tubules show a RSPO1 specific staining. Note the presence of germ cell colonies in XX male with ambiguities (insert) and the presence of isolated germ cells in XX male without ambiguity (arrows for c-Kit staining and insert for RSPO1). Inserts correspond to a 3.0 enlargement of the red rectangles depicted on the same picture. m = mesonephros; i = isthmus between gonad and mesonephros; sc = sub-cortical region. Scale bars: 100 μm. b) Confocal microscopic views of a germ cell colony (10 cells) located in the tunica albuginae of the XX testis (N°450), after RSPO1 immuno-detection. Sale bars: 20 μm. c) Confocal microscopic views of isolated meiotic germ cells in the cortical area of a 60 d*pc *normal ovary, after RSPO1 immuno-detection. Scale bars: 10 μm. b-c) Left picture is a view of one confocal slice (0.36 μm thickness). Middle and right pictures are projected views of the different slices (n = 50) into two different angles, which allow to ascertain the punctuated staining at the cell membrane (as example the same fluorescent point is marked with an arrow in the three views).

To obtain highlight on the function of RSPO1 during goat ovarian development, we studied its cellular and sub-cellular localization at three critical stages of gonad development, 36, 40 and 50 d*pc*, and compared with those of FOXL2 and c-Kit. At the earliest 36–40 d*pc *stages, RSPO1 was found to be mainly localized in the cortical area of the female gonads (illustrated at 40 d*pc*, Fig. 5a). At these stages, FOXL2 positive cells were mainly localized in the ovarian sub-cortical region. Interestingly, the cortical RSPO1 positive area contains the majority of the germ cells that express c-Kit; but RSPO1 appears localized around both somatic and germinal cells (Fig 5a). Sub-cellular localization of RSPO1 was within the cell membrane, resembling those of c-Kit. At 50 d*pc*, the strongest RSPO1 specific signal was found around the germ cells, that are easily recognizable by their round and voluminous nuclei (Fig 5b). Double immunofluorescence experiments carried out for RSPO1 and c-Kit confirm the localization of both proteins on germ cell membranes (Fig. 6a). Moreover, *in situ *hybridization performed on 45 d*pc *ovaries shows that both somatic and germinal cells expressed the *RSPO1 *gene (Fig 6b). Intriguingly, at the 50 d*pc *stage, RSPO1 protein shows a punctuated staining that is not visible at the 40 d*pc *stage (Fig. 5 and Fig. 7). Interestingly at the 50 d*pc *stage, RSPO1 staining appears quite similar to those obtained with anti-WNT4 and anti-β-catenin specific antibodies (Fig. 6c). On another hand, no RSPO1 staining has been observed on male gonads of the same stages, but epithelial cells of the mesonephros has been found positively stained [see Additional file 2].

### RSPO1 localization in XX sex-reversed gonads at 50 d*pc*

In order to get a better understanding of the different levels of *RSPO1 *expression between XX male with ambiguities (N°450) and XX male without ambiguity (N°454) noticed in RT-PCR experiments, RSPO1 protein localization was analyzed on gonadal sections of both fetuses (Fig 7a,7b). AMH and c-Kit detections clearly show that testis from the "pure" XX male is more developed than those of the XX male with ambiguities; seminiferous tubules are more differentiated and delineated and there are more c-Kit positive Leydig cells in testis of 454 compared with those of 450 (Fig 7a). Interestingly, RSPO1 staining specifically remains on isolated germ cell (N°454) or on germ cell colonies (N°450) located in the cortical region, outside the testicular sub-cortical area. Accordingly with the differences noticed on *RSPO1 *mRNA levels, the number of germ cell colonies located outside the seminiferous tubules is clearly increased in the 450 gonad as compared with the 454 one (Fig. 7a). On these germ cell colonies, the punctuated RSPO1 staining remains evident and clearer than in ovaries where the cellular density is higher. Using confocal microscopy, the punctuated staining appears as patches located on the germ cell membranes, with many small patches and one or two big patches (~1 μm) per cell (Fig. 7b). A similar RSPO1 punctuated staining was observed on germ cells lying in the cortical part of normal ovaries, before (50 d*pc*) and during (60 d*pc*) meiosis (Fig. 5b and Fig. 7c).

## Discussion

### RSPO1 protein structure

An intriguing structural feature of all R-Spondin proteins is to display an N-terminal signal peptide and a conserved C-terminal putative nuclear localization signal. Thus, although these proteins have been reported to function as secreted molecules acting on receptors located across the cell membranes [28,29], they might also localize in the nucleus and in fact their predicted sub-cellular localization is in the nucleus [35]. It is thus possible that either the C-terminal basic domain is not a nuclear localization signal but plays a functional role in the secreted proteins, or that R-spondin isoforms lacking the N-terminal signal peptide do exist and play a role in the nucleus. Interestingly, a minor isoform, coding for a protein lacking the signal peptide has been reported in humans but not in mice [17]. Here we show that the goat *RSPO1 *transcript presents an in frame ATG codon located fifteen amino acids downstream of the initiator codon. Remarkably, human *RSPO2*, *3*, *4 *genes also present a second putative initiator codon at positions 16 or 17, and these second ATG are conserved in different mammals (mouse, rat, cow, dog and monkeys).

In order to establish the stability and cellular sub-localization of both putative proteins, we produced and analyzed by cell transfection two constructs with or without the signal peptide. The ATG_1 _construct encodes a secreted RSPO1 protein associated with the cell surface, probably and as previously described by other by binding heparin sulfate proteoglycans on the plasma membrane and extracellular matrix [28,36]. Indeed, we detected goat RSPO1 in the culture medium of transfected COS7 cells only after heparin addition (Fig. 4c). By contrast, the goat RSPO1 protein translated from the second putative initiator codon (ATG_15_) is found in the nucleus of transfected COS7 cells (Fig. 4b), as previously shown for a mouse FLAG-tagged Rspo1 protein [37]. According to this feature, the synthesis of R-spondin proteins without a signal peptide located in the nucleus cannot be excluded. Either these putative isoforms could arise from a translational event that uses the second initiator codon, or from differential splicing events as described for the human *RSPO1 *gene [17]. Nevertheless, a role of these R-spondin proteins within the nuclear compartment remains to be demonstrated *in vivo*. Indeed, in our present studies on goat gonads, RSPO1 protein was never detected in the nucleus.

### *RSPO1 *is not downstream of *FOXL2 *in ovarian differentiation

Homozygous mutations of *RSPO1 *have been found to be responsible for an XX sex-reversed phenotype in human [17]. The only other reported loss-of-function mutation leading to XX sex-reversal in mammals is the PIS mutation in goat. The PIS regulatory mutation leads to a transcriptional silencing of at least three genes in goat ovaries, *FOXL2*, *PFOXic *and *PISRT1 *[16,22]. *FOXL2 *is the unique classical PIS-regulated gene and encodes for a transcription factor responsible for BPES in heterozygous mutated patients (Blepharophimosis Ptosis Epicanthus inversus Syndrome, MIM#110100) [25]. Intriguingly, both *RSPO1*, *FOXL2 *and PIS mutations are also associated with failure of epithelium differentiation at different specific position, palmoplantar hyperkeratosis in *RSPO1*^-/- ^patients, eyelids malformation in *FOXL2*^+/- ^patients and hornless in PIS^-/- ^goats. Another common feature is the absence of obvious testicular phenotype in XY homozygous mutants [16,17,21].

The major objective of this study was to establish if the PIS mutation influence the pattern of expression of *RSPO1 *during ovarian differentiation. By using both RT-PCR and immuno fluorescence, we have shown that transcription of *RSPO1 *is not regulated by *FOXL2 *or other PIS-regulated genes. *RSPO1 *expression is not affected at 40 d*pc *when masculinization occurs in XX sex-reversed gonads. In addition, RSPO1 positive cells are located in the ovarian cortex, a region where *FOXL2 *is not expressed at 40 d*pc*. It is thus possible that either *FOXL2 *and *RSPO1 *belong to two different ovarian pathways, or that *FOXL2 *expression is regulated by* RSPO1*. Mouse carrying null mutations for *Rspo1 *will probably clarify this last point, but until now, as no eyelids phenotype has been shown in human *RSPO1*^-/- ^patients, it seems improbable that *RSPO1 *regulates *FOXL2*. Consequently, the *RSPO1 *anti-testis action is likely to be independent from the *FOXL2 *one, suggesting that different anti-testes genes act in different cell types to ensure proper ovarian differentiation and maintenance. Ottolenghi and collaborators have recently exposed this theoretical view [38], reinforced by the present study and by their recent results on mice carrying null alleles for both *Foxl2 *and *Wnt4 *genes [39].

### Relationships between *RSPO1 *and the *WNT *genes

It has been proposed that R-spondins may synergize with the WNT proteins, both acting through the β-catenin pathway [29]. Among the WNT family, *WNT4 *has been shown to be an important factor for gonad differentiation [14,40,41]. Interestingly in our model, *RSPO1 *and *WNT4 *expressional profiles are similar. Their levels of expression are not decreased in 40 d*pc *XX PIS^-/- ^gonads, and remain detectable in sex-reversed gonads of XX male with ambiguities compared to those of XX male without ambiguity, at 50 or 56 d*pc *[19]. Moreover, WNT4 protein distribution on goat gonads appeared highly similar to that of RSPO1, being present in the cortical area in early 36–40 d*pc *stages (not shown), and mainly associated with the germ cell membranes at the 50 d*pc *stage (Fig. 6c). Although these two genes seem to belong to the same anti-testis pathway, their molecular mechanisms of action could be slightly different. Indeed, a recent study on HEK-293 cells demonstrated that *Rspo1 *regulates Wnt signaling by acting on the co-receptor *LRP6 *levels on the cell surface [42]. Accordingly, no relocation of WNT4 protein has been observed at the germ cell surface (Fig 6c).

### RSPO2 is a candidate gene for ovarian differentiation

Interestingly, we found that another R-spondin gene, *RSPO2 *has a female specific sex-dimorphic expression pattern and that its expression is affected in XX PIS^-/- ^gonads. *RSPO2 *expression level decreases as early as 36 d*pc *and remains low at 40 and 50 d*pc*, a situation highly similar to that observed for the *CYP19 *gene previously shown to be a direct target of *FOXL2 *in goat and tilapia [19,31,43]. These results suggest that *RSPO2 *could represent a direct target of *FOXL2 *in early developing goat ovaries. Although *RSPO2 *ovarian localization and *RSPO2 *promoter studies are needed to assess a putative direct transcriptional activation of *RSPO2 *by *FOXL2*, *RSPO2 *can be considered as a good candidate gene for premature ovarian failure and XX sex-reversal in human.

## Conclusion

The present study brings evidences that *RSPO1 *could have a role correlated with germ cell differentiation and maintenance before and during meiosis. If the first ovarian differentiation failure occurs on the germ cell lineage in human XX *RSPO1*^-/- ^sex-reversed patients, the physiopathology of this disease will be clearly different from those observed in XX PIS^-/- ^sex-reversed goats, for which the primary defect has been localized on supporting cells [19]. By contrast, XX sex-reversal in pigs seems to be linked to a failure of the germ cell maintenance [44] and could result from a mutational event in a gene belonging to the *R-spondin *or *WNT *pathways. Conclusively, it becomes now more evident that different somatic cellular types exist in early differentiating goat ovaries and that different anti-testis pathways should be active in these different cell types in order to ensure proper ovarian differentiation.

## Methods

### Animals

Procedures for handling animals were conducted in compliance with the guidelines for Care and Use of Agricultural Animals in Agricultural Research and Teaching (authorization no. 78-34). All goat fetuses were obtained from horned pregnant females, following hormonal treatments as previously described [19]. Sex-reversed XX males were obtained by crossing two heterozygous polled parents, then testing the absence of the PIS region by PCR as previously described [16]. Day 0 *post-coïtum *corresponds to the day of mating. The genetic sex of all fetuses was determined by PCR amplification of *SRY *and *ZFY/ZFX *genes, on liver genomic DNA [19]. For each fetus, one gonad was frozen in liquid nitrogen for expressional analyses; the other one was fixed for immuno-histological studies.

### RT-PCR

RNA extraction, DNase treatment, and cDNA synthesis were conducted as previously described [16]. One-twentieth (1 μl, corresponding to 0.25 μg of reverse transcribed total RNA) of each RT mix was amplified in 25 μl of PCR reaction by using 0.25 U of Taq polymerase (TaKaRa), 200 μM of each dNTP, and 150 nM of each primer. PCR conditions and primer sequences are given in Table 1. After amplification, 10 μl of each sample were separated by electrophoresis on agarose-TBE gels. All RT-PCR experiments have been done in triplicate.

### Rapid amplification of cDNA ends (5'RACE)

To determine the *RSPO1 *transcriptional start site, four 5' RACE experiments were carried out, each on 5 μg of DNase-treated total RNA purified from 45 or 50 d*pc *goat ovaries (2 samples per developmental stage). Reverse transcription of *RSPO1 *specific RNA was done by using the primer Spondin-2 (Table 1). Reverse transcription assays, then PCR amplifications were performed by using the 5'RACE System from GibcoBRL, according to the manufacturer's instructions. Briefly, two rounds of PCR using nested primers were carried out. The first round of PCR was done with Spondin-3, the second round with either Spondin-6 or Spondin-5 (Table 1). PCR products obtained after the second round of PCR and showing a 106 bp difference in size (which is the length present between the 2 primers used, Spondin-6 and Spondin-5) were cloned. Then, at least 3 independent clones per PCR product were sequenced.

### Genbank accession numbers

All the sequencing was done by MWG-Biotech (Ebersberg – Germany). Genomic DNA sequences are derived from goat BAC sub-clones. *R-spondin *cDNAs were obtained after PCR, then cloned in pGEM^R^-T Easy vector from Promega. All sequences can be found in Genbank with the following accession number: EF486267 (*RSPO1 *mRNA, complete cds), EF486268 (*RSPO2 *mRNA, partial), EF486269 (*RSPO3 *mRNA, partial), EF486270 (*RSPO4 *mRNA, partial), EF486271 (*RSPO1 *gene, 5' sequence).

### Phylogenetic analysis

Goat *RSPO1 *ORF sequence attained in the present study was compared with the four *R-spondin *genes from seven mammalian species (Human, Chimpanzee, Rhesus monkey, Cow, Dog, Mouse and Rat) available on NCBI and Ensembl databases. Sequences comparison and alignment were performed using ClustalW [45] implemented in BioEdit 7.0.5 [46]. Accession numbers are given below. Genetic distances were calculated according to Tamura 3-parameter substitution model with heterogeneous pattern among lineages and uniform rates among sites [47]. A Neighbor-Joining (NJ) tree was built from the genetic distances matrix [48]. Consistency of each node was estimated by bootstrap test after 5000 replicates. Genetic distances and phylogenetic analysis were conducted using MEGA 3.1 [49].

Accession numbers of the sequences used in this phylogeny are as follows: *RSPO1 *(*Homo sapiens*: NM_001038633; *Pan troglodytes*: XM_001169684; *Macaca mulatta*: ENSMMUT00000011770; *Bos taurus*: ENSBTAT00000005002; *Canis familiaris*: ENSCAFT00000005262; *Mus musculus*: NM_138683; *Rattus norvegicus*: XM_233520); *RSPO2 *(*Homo sapiens*: NM_178565; *Pan troglodytes*: XM_001134914; *Macaca mulatta*: XM_001089438; *Bos taurus*: ENSBTAT00000018083; *Canis familiaris*: ENSCAFT00000001085; *Mus musculus*: NM_172815; *Rattus norvegicus*: XM_576261); *RSPO3 *(*Homo sapiens*: NM_032784; *Pan troglodytes*: XM_001166327; *Macaca mulatta*: XM_001106900; *Bos taurus*: NM_001076034; *Canis familiaris*: XM_533492; *Mus musculus*: NM_028351; *Rattus norvegicus*: XM_574288); *RSPO4 *(*Homo sapiens*: NM_001029871; *Pan troglodytes*: XM_525242; *Macaca mulatta*: XM_001112556; *Bos taurus*: ENSBTAT00000029170; *Canis familiaris*: ENSCAFT00000010997; *Mus musculus*: NM_001040689; *Rattus norvegicus*: XM_575261).

### Plasmids construction, COS7 cell culture conditions and transfection assays

Two different goat *RSPO1 *cDNAs were obtained after RT-PCR on a 50 d*pc *ovary with primers (SpondinATG1bis or SpondinATG2 and SpondinTGA). Both, (pSG5-ATG_1_, pSG5-ATG_15_), were inserted in the pSG5 vector (Stratagene). COS7 cells were cultured in Dulbecco's modified Eagle's medium and Ham's F-12 medium (Eurobio, Courtaboeuf, France) containing 10% (v/v) heat-inactivated foetal calf serum, penicillin (100 U/ml), streptomycin (100 μg/ml) and L-glutamine (300 μg/ml), in a 5% CO2 atmosphere at 37°C. For transfection, 2 × 10^5 ^cells were seeded in 60 mm diameter petri dishes and transfected the following day using FuGENE 6 reagent (Roche), according to the manufacturer's instructions. Before transfection, the medium was changed by the serum free Dulbecco's modified Eagle's medium and Ham's F-12 medium added with soluble heparin (50 μg/ml, Sigma H-1027). The amount of transfected DNA was 4 μg for either expression vectors (pSG5-ATG_1_, pSG5-ATG_15_). Seventy-two hours after transfection, cells were harvested by scraping in 1 ml phosphate-buffered saline (PBS), pelleted by centrifugation and rapidly frozen at -80°C until use. The culture mediums were recovered and concentrated with a VIVASPIN 6 ml Concentrator (VIVASCIENCE).

### Cytosolic and nuclear protein extracts

The cell pellets were recovered in extraction buffer (20 mM Tris-HCl (pH 8), 137 mM NaCl, 2.7 mM KCl, and 10% glycerol) containing protease and phosphatase inhibitors. Cell lysis was performed by adding Nonidet P-40 (*IGEGAL CA-630*) at a final concentration of 0.5% (v/v) to the cell suspensions. After homogenization and a brief incubation at 4°C for 5 min, the mixtures were gently layered onto 6 ml separation buffer A (10 mM HEPES (pH 7.7), 25 mM KCl, 2 mM EDTA, 0.5 mM EGTA (pH 8), containing 1 M sucrose). After centrifugation (15 min. at 3500 RPM) the supernatant fraction containing cytosolic protein extract was recovered. The pellet containing nuclear protein extract were resuspended in buffer B (20 mM HEPES (pH 7.7), 1.5 mM MgCl_2_, 0.2 mM EDTA, 25% glycerol), then cleared by addition of 4 M NaCl (1/10 final volume), mixed gently on a rotating wheel (30 min. at 4°C), and centrifugated (30 min. at 12,000 rpm). Finally supernatants containing nuclear extracts were frozen at -80°C until use. Proteins were quantified with a BCA protein assay kit (Pierce Chemical Co., Bezons, France) according to the manufacturer's protocol with BSA as standard.

### Western Blot

Forty μg of protein extracts (cytosolic, nuclear or culture medium) were boiled in Laemmli buffer and subjected to SDS-polyacrylamide gel electrophoresis (SDS-PAGE). Proteins were then transferred onto nitrocellulose membranes (Hybond ECL, GE Healthcare). Membranes were blocked (1 h at room temperature) in PBS with 10% powdered milk, 0.3% Tween 20, then incubated with a primary antibody anti-RSPO1 (1/5000, overnight at 4°C) [30]. After 3 washes in PBS, 0.3% Tween 20, membranes were incubated with the secondary antibody anti-rabbit coupled to peroxidase (1/6000, 40 min. at room temperature; Jackson Immunoresearch Lab.). After 4 washes, the targeted proteins were revealed by peroxidase activity and detected by chemiluminescent (SuperSignal West Pico, PIERCE).

### Immuno-fluorescence on COS7 cells

COS7 cells were grown on glass coverslips, then fixed in the culture petri dishes with 4% paraformaldehyde in phosphate saline buffer (PBS) for 10 min. After 4 washes in PBS, samples were incubated 30 min. with NH_4_Cl (50 mM) in PBS, then permeabilized with 0.5% saponine, 1% BSA in PBS for 1 h. Samples were incubated overnight at 4°C with the RSPO1 (1/300) and PDI (1/200) primary antibodies [30,34]. After 3 washes in PBS, samples were incubated with the secondary antibodies, anti-rabbit IgG coupled with FITC (1/200, Vector) and anti-mouse IgG coupled with TRITC (1/400, Jackson Immunoresearch lab.) for 45 min at room temperature. Samples were washed in PBS and mounted in Vectashield mounting medium with DAPI (VECTOR). Cells were observed with a Leica DMRB epifluorescence microscope. Acquisition was performed using a DP50 CCD camera (Olympus).

### Immuno-fluorescence on goat ovarian sections

Freshly dissected gonads were fixed 1 h in 4% paraformaldehyde in PBS at 4°C. After washes in PBS with increasing concentrations of sucrose (0, 12%, 15%, and 18%), tissue specimens were embedded in Jung Tissue Freezing Medium (Leica Instruments) and frozen at -80°C. Cryosections (7 μm thick) were obtained and stored at -80°C. After thawing, sections were washed 10 min. in PBS, and quenched in 50 mM NH_4_Cl for 30 min. in PBS, then blocked for 1 h in blocking buffer (1% BSA, 0.5% Saponine in PBS). Sections were incubated overnight at 4°C with the primary antibodies anti-RSPO1 (1/300) [30]; anti-c-Kit (1/100, Santa Cruz Biotechnology), anti-FOXL2 (1/500) [50], anti-AMH (1/50) [51], anti-WNT4 (1/500; M-70, Santa Cruz Biotechnology) or anti β-catenin (1/1000) [52] diluted in fresh blocking buffer. After 3 washes, sections were incubated for 45 min. at room temperature with the secondary antibody anti-rabbit IgG coupled with FITC (1/200, VECTOR), then washed 3 times and mounted. For RSPO1/c-Kit double-staining, immuno-detection were done sequentially with an incubation step of 1 h in blocking buffer added with 10% goat serum between each staining. The RSPO1 staining was done first and revealed with anti-rabbit IgG coupled with FITC (1/200, VECTOR). The c-Kit staining was revealed with anti-rabbit IgG coupled with TRITC (1/200, Jackson Immunoresearch lab.). Slides were mounted in Vectashield mounting medium with DAPI (VECTOR) or with propidium iodide (VECTOR) and observed with a Leica DMRB epifluorescence microscope coupled to a DP50 CCD camera (Olympus). Confocal microscopy was performed with a LSM510 equiped with a HRm Axiocam CCD camera (Carl Zeiss, Germany).

### In situ hybridization

Two cDNA fragments located in the 3'-untranslated region of *RSPO1 *mRNA were obtained by RT-PCR on goat ovaries with primers Spondin12/13 and HIS-RSPO1-1F/2R (Table 1), then subcloned into pGEM^R^-T Easy vector. Riboprobes were generated by transcription in the presence of digoxygenin-labeled deoxy-UTP and the appropriate SP6 or T7 RNA polymerase. Then, in situ hybridization was performed as previously described [31].

## Abbreviations

AMH: Anti-Müllerian hormone; BPES: Blepharophimosis ptosis epicanthus inversus syndrome; CYP19: Cytochrome P450 aromatase; d*pc*: days *post-coïtum*; FGF9: Fibroblast growth factor 9; FOXL2: Forkhead box gene family L member 2; FZD/LRP: Frizzled/LDL receptor-related protein; HMG: High mobility group; PFOXic: Promoter FOXL2 inverse complementary; PIS: Polled intersex syndrome; PISRT1: PIS-regulated transcript number 1; POF: Premature ovarian failure; PPK: Palmoplantar hyperkeratosis; RACE: Rapid amplification of cDNA ends; RT-PCR: Reverse transcriptase polymerase chain reaction; SCC: Squamous cell carcinoma; SOX9: Sry-related HMB-box gene 9; SRY: Sex-determining region of Y chromosome; TCF: T-cell factor; WNT: Wingless related MMTV integration site.

## Authors' contributions

AK and MP carried out immunohistology. AK carried out plasmid constructs and cell transfection. IP and LR carried out expressional studied. IP realized sequence alignment and phylogenic analyses. PP and OR participated in the design of the study. KAK produced the Nu-206 anti-RSPO1 antibody. GC and EP conceived of the study and participated in its design. EP managed the study and drafted a first version of the manuscript. MP, PP, OR and GC improved the manuscript. All authors read and approved the final manuscript.

## Supplementary Material

Additional file 1 *RSPO1 *RT-PCR analyses on goat gonads and mesonephroi. The data show that (i) *RSPO1 *ovarian transcripts mainly start at the transcriptional start site determined by RACE; (ii) all *RSPO1 *ovarian transcripts are with exon 4; (iii) *RSPO1 *is expressed in mesonephroi. Click here for file

Additional file 2 RSPO1 immuno-detection on a male gonad and on mesonephros. The data show that RSPO1 is not detected on a 50 d*pc *male gonad, and that a specific RSPO1 staining is visible on different epithelial cells of a 50 d*pc *male mesonephros. Click here for file
