# Supplementary material for: R-spondin1 and FOXL2 act into two distinct cellular types during goat ovarian differentiation
Source: BMC Dev Biol. 2008 Apr 2;8:36. doi: 10.1186/1471-213X-8-36 (PMC2329615; doi:10.1186/1471-213X-8-36)
Supplement: Additional file 1 — RSPO1 RT-PCR analyses on goat gonads and mesonephroi. The data show that (i) RSPO1 ovarian transcripts mainly start at the transcriptional start site determined by RACE; (ii) all RSPO1 ovarian transcripts are with exon 4; (iii) RSPO1 is expressed in mesonephroi. [file 1471-213X-8-36-S1.ppt]

## Slide 1
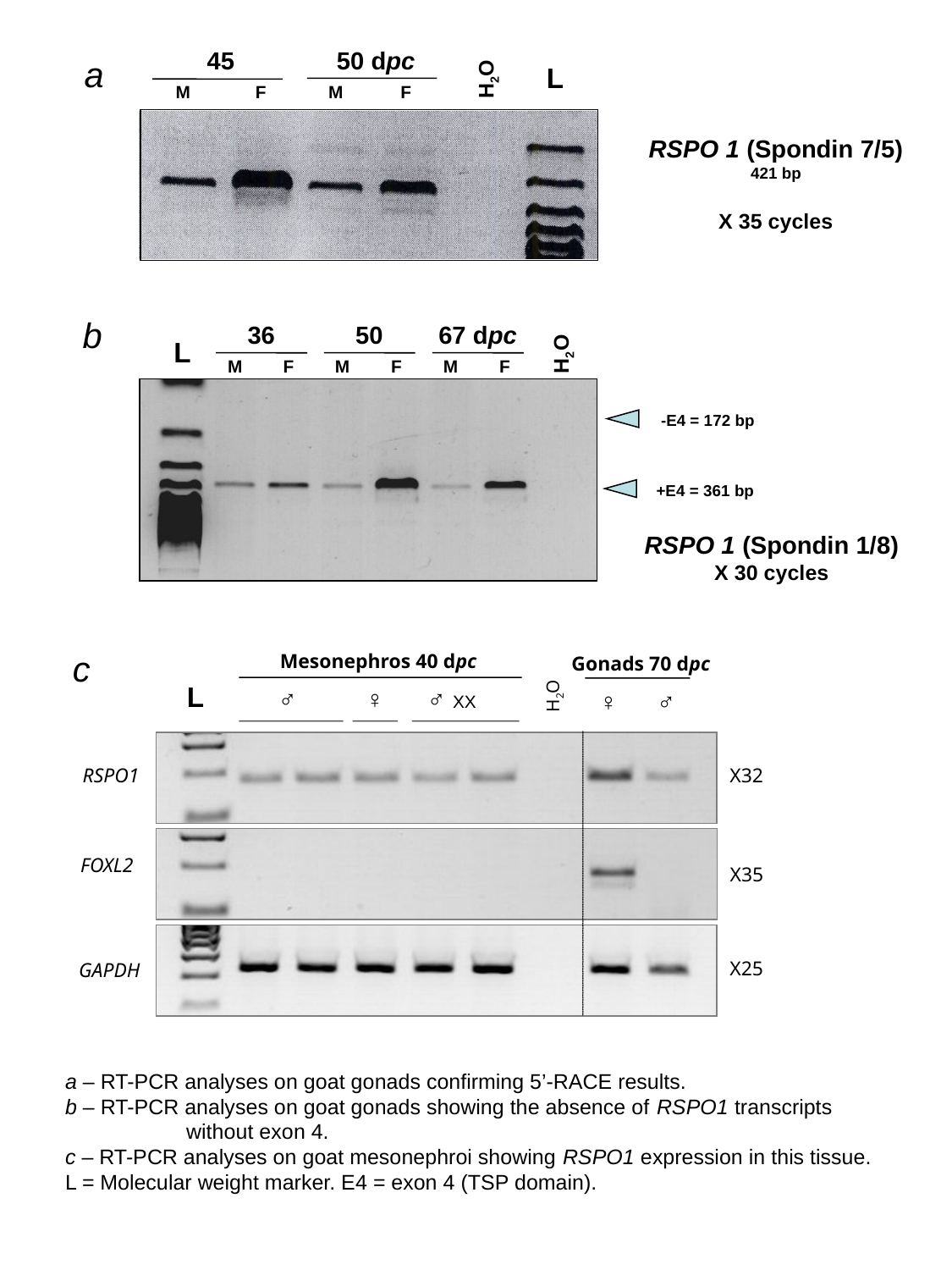

50 dpc
45
a
L
H2O
M
F
M
F
RSPO 1 (Spondin 7/5)
421 bp
X 35 cycles
b
36
50
67 dpc
L
H2O
M
F
M
F
M
F
-E4 = 172 bp
+E4 = 361 bp
RSPO 1 (Spondin 1/8)
X 30 cycles
c
Mesonephros 40 dpc
Gonads 70 dpc
L
♂
♀
♂ XX
H2O
♀
♂
RSPO1
X32
FOXL2
X35
X25
GAPDH
a – RT-PCR analyses on goat gonads confirming 5’-RACE results.
b – RT-PCR analyses on goat gonads showing the absence of RSPO1 transcripts
	without exon 4.
c – RT-PCR analyses on goat mesonephroi showing RSPO1 expression in this tissue.
L = Molecular weight marker. E4 = exon 4 (TSP domain).
