# Supplementary material for: R-spondin1 and FOXL2 act into two distinct cellular types during goat ovarian differentiation
Source: BMC Dev Biol. 2008 Apr 2;8:36. doi: 10.1186/1471-213X-8-36 (PMC2329615; doi:10.1186/1471-213X-8-36)
Supplement: Additional file 2 — RSPO1 immuno-detection on a male gonad and on mesonephros. The data show that RSPO1 is not detected on a 50 dpc male gonad, and that a specific RSPO1 staining is visible on different epithelial cells of a 50 dpc male mesonephros. [file 1471-213X-8-36-S2.ppt]

## Slide 1
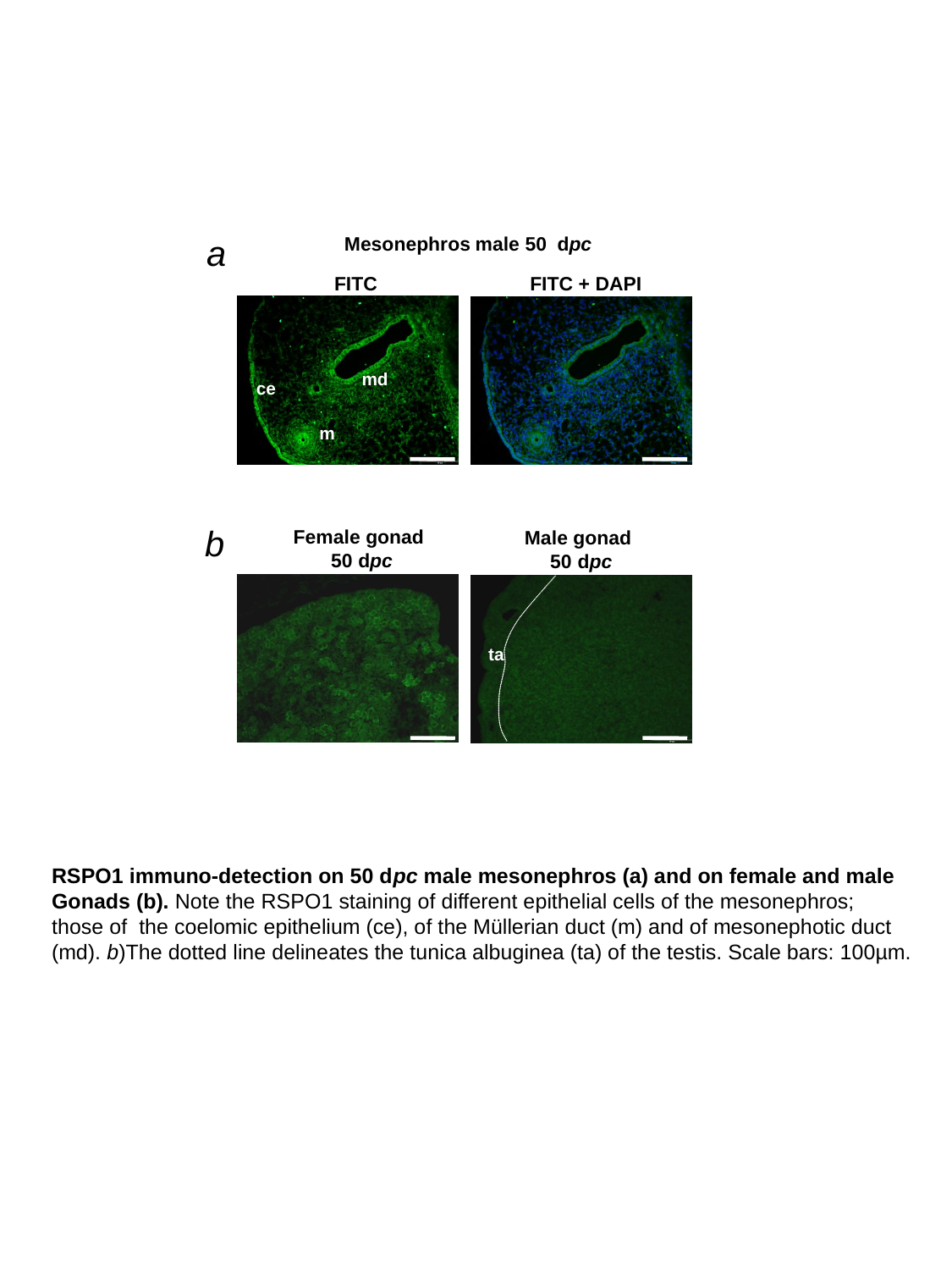

a
Mesonephros
male 50
dpc
FITC
FITC + DAPI
md
ce
m
b
Female
gonad
Male
gonad
50 d
pc
50
d
pc
ta
RSPO1 immuno-detection on 50 dpc male mesonephros (a) and on female and male
Gonads (b). Note the RSPO1 staining of different epithelial cells of the mesonephros;
those of the coelomic epithelium (ce), of the Müllerian duct (m) and of mesonephotic duct
(md). b)The dotted line delineates the tunica albuginea (ta) of the testis. Scale bars: 100µm.
